# Profile of Whole Body Electromyostimulation Training Users—A Pilot Study

**DOI:** 10.3390/ijerph19084711

**Published:** 2022-04-13

**Authors:** Luiz Rodrigues-Santana, Hugo Louro, Ángel Denche-Zamorano, Alejandro Vega-Muñoz, Nicolás Contreras-Barraza, Jose Carmelo Adsuar

**Affiliations:** 1 Faculty of Sport Science, University of Extremadura, 10003 Cáceres, Spain; 2 Sport Sciences School of Rio Maior, Research Center in Sport Science, Health and Human Development, 5000-801 Vila Real, Portugal; hlouro@esdrm.ipsantarem.pt; 3 Life Quality Research Center, 2040-413 Santarém, Portugal; 4 Promoting a Healthy Society (PHeSo), Faculty of Sport Science, University of Extremadura, 10003 Cáceres, Spain; andeza04@alumnos.unex.es (Á.D.-Z.); jadssal@unex.es (J.C.A.); 5 Public Policy Observatory, Universidad Autónoma de Chile, Santiago 7500912, Chile; alejandro.vega@uautonoma.cl; 6 Facultad de Economía y Negocios, Universidad Andres Bello, Viña del Mar 2531015, Chile; nicolas.contreras@unab.cl

**Keywords:** WB-EMS, fitness users, physical activity

## Abstract

(1) Introduction: Whole Body Electromyostimulation is a technological and time efficient personal training practiced all over the world. With the increase of practitioners in the last 10 years, the need to study more about practitioners has arisen, so this pilot study aims to trace a user profile of this method through the analysis of socio-demographic data for a better understand of the profile of people looking for this type of training to improve the effectiveness of the intervention and develop programs that are in accordance with the motivation of practitioners. (2) Methods: 270 users from 5 countries answered an online questionnaire with socio-demographic questions. Data were treated using descriptive statistics. Possible differences between sexes and between groups were analyzed by means of non-parametric statistical tests: Mann–Whitney U-test (continuous variables); in addition to studying possible dependence relationships and differences between proportions, using the Chi-square statistic with pairwise z-test using the Bonferroni correction (categorical variables). (3) Results: Middle-aged women are the main user of this type of training. The majority of WB-EMS users do another type of physical activity with significant difference between men and woman (*p* < 0.05) men are more active than women. Weight loss, health and wellness and muscle mass increase are the main goals of the WB-EMS users. There are significant differences in weight loss and rehabilitation between genders (*p* < 0.05). Women look much more than men to lose weight and men look more than women to rehabilitation. (4) Conclusions: The user profile is a physically active woman, aged 35–49 years, with normal weight and high educational level, who carries out twice weekly full body electrostimulation training with the goals of weight loss, health and/or wellness and muscle mass gain.

## 1. Introduction

Whole Body Electrostimulation (WB-EMS) is a novel, attractive and time efficient training method for physical fitness and rehabilitation [1] which started to be popular in Europe in the last decade and is now present all over the world [2]. The method consists of applying electrical myostimulation throughout the human body, activating up to 8–10 different muscles groups (e.g., quadriceps, hamstrings, glutes, dorsal, chest, abdominal, biceps and triceps), while physical exercises are performed. This combination of voluntary contraction superimposed with the involuntary muscle contraction (the electrical stimulation) should provoke a potential additional gain in the physiologic effects leading to improvements of muscular power, strength or endurance in case of regular application [3].

The WB-EMS has been touted as a more attractive but also more expensive alternative to conventional exercise. In recent years, the use of technology has been a trend in world of fitness, being at the top of user preferences according the American College of Sports Medicine [4,5,6,7].

The investigation in the WB-EMS field was increasing last years with interventions performed with different types of populations. A meta-analysis composed by five controlled trials with low back pain people observed significant improvements in pain intensity in the WB-EMS group training [8]. A mini-meta-analysis of five randomized controlled trial concluded that WB-EMS is a feasible complementary training stimulus for performance enhancement [9]. A systematic review performed with twenty-three articles found that WB-EMS groups significantly improves muscle mass and function while reducing fat mass and low back pain [1]. Some studies demonstrate the effects of the WB-EMS training on special populations such as sarcopenic obesity [10], cardiac [11], post-menopause, elderly [12,13,14] or cancer [15,16,17,18,19], while other studies show effects on healthy populations [20,21,22] and even on athletes [9,23,24,25,26]. Gender has also been the subject of studies, with some scientific works carried out investigated effects only in woman [27,28] others only in men [29].

The frequency of weekly use also does not meet consensus among the scientific community. There are studies conducted with 1 weekly session [30,31,32], 1.5 (3 session in two weeks) [12,33] and 2 times a week sessions [34,35]. The positive effects demonstrated by WB-EMS range from rehabilitation and pain relief [26,36,37], change in body composition [21,38,39,40], increase in muscle mass [41,42], strength [22,25,43] and endurance [44,45,46].

In the last decade there has been a large increase in the number of brands and equipment manufacturers, as well as in the number of professionals and training studios specialized in this training method. Hence, it becomes important to know the profile of people looking for this type of training to improve the effectiveness of the intervention [47] and develop programs that are in accordance with the motivation of practitioners, to develop actions to attract profiles with low use and to know the changes that may occur in users over time. It is known that socio-demographic and motivational aspects are related not only to the beginning of a certain physical activity but also to its abandonment, being crucial to study adherence [48].

Some studies have documented a relationship between motivation and sociodemographic variables, namely gender and age [49,50]. Younger people seem to have as motivation issues more related to enjoyment and aesthetic related to body composition [51,52], while health and well-being appear as the main factor for the practice of physical exercise as age increases [53,54,55]. The same authors also found relation in the gender, men are more concerned with enjoying and socializing while women are more concerned with appearance and health [53,55]. The variable level of study has also been related to the practice of physical activity, as the level of study increases, the practice of physical exercise is also greater [55,56].

By our knowledge there is no study describing the socio-demographic profile and motivation of the users of WB-EMS. Therefore, the aim of this study is to determine the profile of the users who seek this type of training method and make it a routine in their daily lives. In this study we center ourselves in the characteristics that define the personal, social and sports profile of the users: body mass index, group age, marital status, level of studies, training frequency and motivation (goals).

## 2. Materials and Methods

### 2.1. Study Design

The study was conducted as a cross sectional descriptive study. It has been established profiles of the Whole Body Electromyostimulation training users.

### 2.2. Participants

In all, 270 WB-EMS users from 5 different countries participated in this study; the 5 countries were chosen for convenience and ease of getting answers from the users. The questionnaire in digital format (QR code) was sent to the studios that agreed to participate. The manager of each studio sent the questionnaire and asked all clients to participate in the study. The inclusion criteria were being over 18 years old and having practiced WB-EMS for at least 1 month. All subjects participated voluntarily and provided written informed consent for inclusion in the study. The study was conducted in accordance with the Declaration of Helsinki, and approved by Ethics Committee of University of Extremadura, register number 157/2021 on 29 September 2021.

### 2.3. Questionnaire

The questionnaire consisting of 14 direct answer questions on personal and sociodemographic data [52]: gender, weight, height, marital status, level of study, training goals, training frequency and habits and place. It was anonymous and confidential and was designed for online collection of data.

From weight and height, the BMI variable is created, with the formula: kg/m^2^. From this, the BMI Group categorical variable was created, making the following groups: Underweight (≤18.5), Normal (between 18.5 to 24.99), Overweight (between 25 to 30) and Obese ≥ 30. From the age onwards, the Age Group variable was created: 18–34, 35–49, 50–64 and ≥65. The Academic studies variable was created, grouping in “university” (degree’s, master’s or doctoral) and “no university” (not have attended university).

### 2.4. Procedure

Participants were asked to complete a questionnaire before or after the training session. People from five different countries (Portugal, Brazil, Hungary, Belgium and Indonesia) participated in this study, Portugal and Brazil answered a Portuguese version and the other three countries answered an international version in English. The questionnaire was made available online via QR code at the entrance of the studios or sent a link via phone message with prior authorization. Participants asked on their own device (smartphone or tablet) alone. The average time taken to answer the questionnaire was 10 min.

### 2.5. Statistical Analysis

The normality of the data was tested by Kolmogorov–Smirnov test. Continuous variables were presented as median and interquartile range. Categorical variables were presented as absolute and relative frequencies. Differences between sexes and between groups were analyzed by means of non-parametric statistical tests: Mann–Whitney U-test (continuous variables). In addition to studying possible dependence relationships and differences between proportions, using the Chi-square statistic with pairwise z-test using the Bonferroni correction (categorical variables). All analyses were performed using a level of significance <0.05. IBM SPSS (V.22, IBM Corporation, New York, NY, USA) was the statistical software used.

## 3. Results

The result of the Kolmogorov–Smirnov test did not show evidence to assume normality in any of the variables studied.

The descriptive statistics generated by the total of the sample (Table 1) revealed that the profile of the WB-EMS user is a middle-aged woman (35–49), with normal weight, active (59% do another type of physical activity apart from WB-EMS training) with university studies, who seeks one of the following three goals: weight loss, increasing muscle mass or improving health and wellness. On the other hand, we can also profile the male user, it is a little younger (33% vs. 27.6% between 18–34 years), also with university studies (85%) and more active than women’s (76.2% vs. 59.8% *p* < 0.05 from pairwise z-test). About the goals, they look more for muscle mass increase (*p* < 0.05 from pairwise z-test) and health.

In the Table 2 we can see the relation between the socio-demographic and the training goal variables. We did not find significant differences between any variable. However, we managed to establish a relationship of dependence between objective and age.

In comparison with the weekly training frequency (Table 3), there are no significant differences with any of the socio-demographic variables studied. Neither is there any dependency relationship with any variable and frequency. With more knowledge about the profile users, we can better adapt communication and training programs to improve retention and acquisition of new practitioners and adapt the market to the users’ needs.

**Figure 1:**
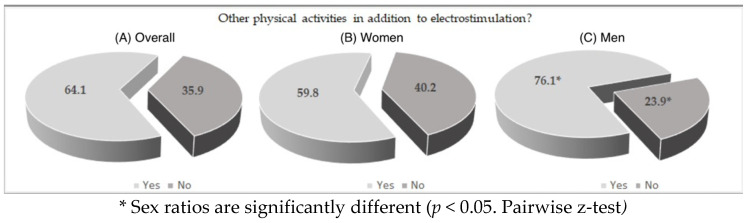
Percentage of users who do or do not practice another activity besides WB-EMS training. (**A**) All samples; (**B**) women’s group; (**C**) men’s group.

**Figure 2:**
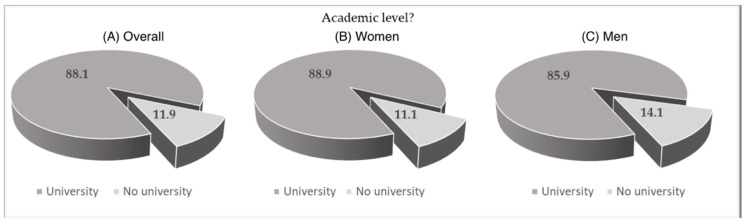
Academic level of WB-EMS users. (**A**) All samples; (**B**) women’s group; (**C**) men’s group.

Looking to the charts below (Figure 1), we can observe that, regardless of gender, the vast majority of these users do another type of physical activity (64.1%) with significant difference between men and woman (*p* < 0.05 from pairwise z-test). Regarding the academic level we can see (Figure 2) that we can see that 8 out of 10 users have higher education level (at least a degree) there are no significant differences between the genders.

**Figure 3:**
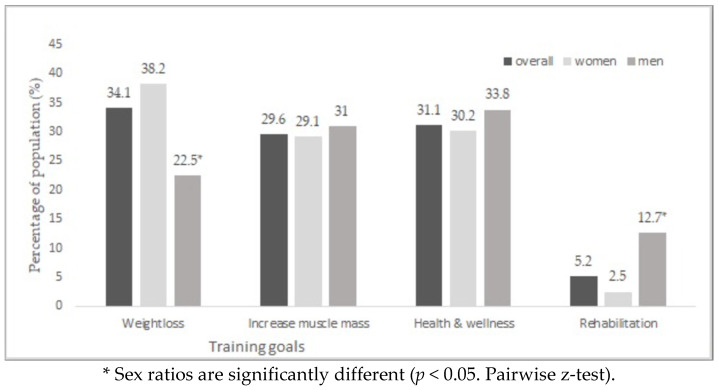
Training goals by gender.

Regarding the motivation to practice (training goals), weight loss, health and wellness and increase in muscle mass are the main reasons for practice respectively. In the comparison between genders, there are significant differences in weight loss and rehabilitation (*p* < 0.05 from pairwise z-test). Women look much more than men to lose weight and men look more than women to rehabilitation with WB-EMS (Figure 3).

## 4. Discussion

The result showed that those looking for this type of training more are middle-aged women. The greater demand for Personal Trainer services by women may be related to a cause of safety and guarantee of better results [57]. The Whole Body Electromyostimulation training is advertised by brands as very effective in weight loss and body remodeling may also be a reason why it is more sought after by women, as several studies point out that improving appearance, controlling weight, socializing and self-esteem are among the main reasons for physical activity [58,59,60]. On the other hand, the little external load (weights) needed in WB-EMS training may be a possible reason for less demand by men, since weight training (bodybuilding) and muscle mass increase be a more common goal in men.

The dependence between objective and age is in agreement with other studies found, older people seek health and well-being [61,62,63,64,65] and younger people seek changes in body composition (losing weight or gaining muscle mass), image improvement and social recognition [66,67,68]. In this study we can observe that in the first age groups (18 to 49 years old), more than 50% of users seek WB-EMS training to change their body composition (lose weight or increase muscle mass) while in the older age groups (over 50 years old) there is a tendency (more than 50%) to be motivated by health or rehabilitation. About effectiveness of this training method according to the objectives sought by both younger and older people, a systematic review and meta-analysis performed with 16 studies demonstrated the effectiveness of WB-EMS training in change body composition [40] as well as other randomized controlled trail studies have shown that WB-EMS can be an alternative to fight old age diseases such as sarcopenia and osteoporosis [13,14].

The relationships between socio-demographic variables and training goals results indicate that people who are overweight or obese (BMI > 25) seek to lose weight. We have the case of only 1 person with a low BMI who referred to aim to lose weight, this case may be associated with some pathology related to image distortion. Users with a normal BMI are mainly looking to increase muscle (41.9%) mass and improve health and wellness (36.5%). The civil status and lever of studies, and whether or not to do any other physical activity seems to have nothing to do with training goals.

Regarding the number of sessions per week, we did not find a relationship with the type of objective, but we can mention that the majority (53%) do two session a week. Overweight users are the ones with the highest percentage who also do two session per week (75%). Divorced and widowed mostly do only one session a week (53.6%) while married and single people mostly do two weekly sessions.

We observed that most WB-EMS users (64%) do another activity in addition to training with electrostimulation, which contrasts a little with the reputation that this method has gained as a type of physical activity for those who do not like to train.

Although there are some studies relating the level of studies with the practice of physical activity that report that people with a higher level of education give more importance [69] to and do more physical activity than people with a lower level of education [56] we did not found any difference between the WB-EMS users. We can observe that the vast majority of users have university studies, which can be explained by the price of the sessions. A possible explanation for this observation may be that people with higher education with a higher socio-economic level will have more access and can afford this type of training.

Weight loss, health and well-being and increase muscle mass, in that order, were the main goals pointed out by WB-EMS users of this method, which is not new and is in line with the results of some studies that point to being fit and healthy as the main reasons for physical exercise [70,71].

From a practical point of view, this study helps us to understand WB-EMS users in order to better adapt communication and training programs to improve retention and acquisition of new practitioners. This type of studies has been important to know the different profiles and to adapt the market to the users’ needs [50,72]. Further than that nowadays, where the numbers of sedentary lifestyle and the consequent deaths that could be avoided with more exercise [73,74], it is essential to understand the profile and motivations of those who practice to promote more effective programs.

As for the limitations of this study, we have to point out that due to the fact that we have a small and convenient sample, we cannot say that the sample is representative. We collected data from five different countries, with different cultures that may have an influence on the profile traced. The data collection, when conducted through an online questionnaire, may have made it difficult for older people to participate. More studies will be needed with larger sample to better be able to trace the user profile and study its relationship with socio-demographic variables. We suggest further studies about the barriers and benefits of training with electrostimulation as well as to study the main reason why users choose to do this methodology to the detriment of others.

## 5. Conclusions

The results of this pilot study indicate that in these five countries the most common user profile is a physically active woman, aged 35–49 years, with normal weight and high educational level, who carries out twice weekly full body electrostimulation training with the goals of: weight loss, health and/or wellness and muscle mass gain.

Training goals are related to gender, age group and BMI condition. Weight loss is the main goal for young adults, women and people who are overweight or obese. Gaining muscle mass is the main goal among young people. Health is the main objective for men and especially for older adults.

With the accomplishment of this pilot study, we also concluded that it is necessary to carry out more research in this area, with a larger and representative sample, to confirm the evidence found in this study and obtain conclusions that may be representative. It is necessary to go further along this line of investigation, since there is a lack of knowledge about the profiles of the users who train in whole body electrostimulation centers, as well as the motivations that lead them to use this kind of training.

## Figures and Tables

**Table 1:** Descriptive analysis and comparation between sex.

Variables	Overall = 270 Mdn (IQR)	Women = 199 Mdn (IQR)	Men = 71 Mdn (IQR)	*p*
Age (Years)	39 (16)	39 (13)	39 (20)	0.487
Weight (kg)	68.0 (17.5)	64.0 (14.0)	80.0 (16.0)	<0.001
Height (m)	1.73 (0.13)	1.64 (0.09)	1.76 (0.09)	<0.001
BMI (kg/m^2^)	24.5 (4.5)	23.8 (4.8)	25.9 (5.0)	<0.001
Experience (months)	6 (12)	6 (12)	6 (15)	0.580
Age groups	Overall *n* (%)	Women *n* (%)	Men *n* (%)	*p* *
18–34 years	79 (29.3)	55 (27.6)	24 (33.8)	<0.37
35–49 years	127 (47.0)	101 (50.8)	26 (36.6) *	
50–64 years	56 (20.7)	40 (20.1)	16 (22.5)	
65+ years	8 (3.0)	3 (1.5)	5 (7.0) *	
BMI groups	Overall *n*(%)	Women *n* (%)	Men *n* (%)	*p* *
Underweight	4 (1.5)	4 (2.0)	0 (0.0)	<0.006
Normal	148 (55.0)	120 (60.6)	28 (39.4) *	
Overweight	90 (33.5)	56 (28.3)	34 (47.9) *	
Obese	27 (10.0)	18 (9.1)	9 (12.7)	
Civil status	Overall *n* (%)	Women *n* (%)	Men *n* (%)	*p* *
Singles	108 (40.0)	77 (38.7)	31 (43.7)	0.139
Married	134 (49.6)	97 (48.7)	37 (52.1)	
Divorced/Widowers	28 (10.4)	25 (12.6)	3 (4.2) *	
Level of studies	Overall *n* (%)	Women *n* (%)	Men *n* (%)	*p* *
No university	32 (11.9)	22 (11.1)	10 (14.1)	0.498
University	238 (88.1)	177 (88.9)	61 (85.9)	
Training goals	Overall *n* (%)	Women *n* (%)	Men *n* (%)	*p* *
Weightloss	92 (34.1)	76 (38.2)	16 (22.5) *	<0.002
Increase muscle mass	80 (29.6)	58 (29.1)	22 (31.0)	
Health and wellness	84 (31.1)	60 (30.2)	24 (33.8)	
Rehabilitation	14 (5.2)	5 (2.5)	9 (12.7) *	
Sessions per week	Overall *n* (%)	Women *n* (%)	Men *n* (%)	*p* *
1	103 (38.1)	69 (34.7)	34 (47.9) *	0.144
2	144 (53.3)	112 (56.3)	32 (45.1)	
3+	23 (8.5)	18 (9.0)	5 (7.0)	
Other physical activities	Overall *n* (%)	Women *n* (%)	Men *n* (%)	*p* *
Yes	173 (64.1)	119 (59.8)	54 (76.1) *	<0.014
No	97 (35.9)	80 (40.2)	17 (23.9) *	
Equipament	Overall *n* (%)	Women *n* (%)	Men *n* (%)	*p* *
With wire	78 (28.9)	54 (27.1)	24 (33.8)	0.287
Wireless	192 (71.1)	145 (72.9)	47 (66.2)	

*n* (participants); % (percentage); Mdn (median); IQR (interquartile range); *p* * (*p*-value from Chi-square test); *p* (*p*-value from Mann–Whitney U test); BMI (body mass index, kg/m^2^); Underweight (BMI < 18.5); Normal (BMI ≥ 18.5 and <25); Overweight (BMI ≥ 25 and <30); Obese (BMI ≥ 30); University (university studies); No university (no university studies); * (sex ratios are significantly different).

**Table 2:** Relationships between socio-demographic variables and training goals.

Age Groups	Weightloss *n* (%)	Increase Muscle Mass *n* (%)	Health and Wellness *n* (%)	Rehabilitation *n* (%)	*p*
18–34 years	25 (31.6)	29 (36.7)	25 (31.6)	0 (0.0)	<0.001
35–49 years	62 (48.8)	34 (26.8)	28 (22.0)	3 (2.4)	
50–64 years	5 (8.9)	15 (26.8)	28 (50.0)	8 (14.3)	
65+ years	0 (0.0)	2 (25.0)	3 (37.5)	3 (37.5)	
BMI groups	Weightloss *n* (%)	Increase muscle mass *n* (%)	Health and wellness *n* (%)	Rehabilitation *n* (%)	*p*
Underweight	1 (25.0)	0 (0.0)	3 (75.0)	0 (0.0)	<0.001
Normal	26 (17.6)	62 (41.9)	54 (36.5)	6 (4.1)	
Overweight	42 (46.7)	18 (20.0)	22 (24.4)	8 (8.9)	
Obese	22 (81.5)	0 (0.0)	5 (18.5)	0 (0.0)	
Civil status	Weightloss *n* (%)	Increase muscle mass *n* (%)	Health and wellness *n* (%)	Rehabilitation *n* (%)	*p*
Singles	40 (37.0)	35 (32.4)	31 (28.7)	2 (1.9)	0.110
Married	47 (35.1)	36 (26.9)	40 (29.9)	11 (8.2)	
Divorced/Widowers	5 (17.9)	9 (32.1)	13 (46.4)	1 (3.6)	
Level of studies	Weightloss *n* (%)	Increase muscle mass *n* (%)	Health and wellness *n* (%)	Rehabilitation *n* (%)	*p*
No university	11 (34.4)	13 (40.6)	7 (21.9)	1 (3.1)	0.428
University	81 (34.0)	67 (28.2)	77 (32.4)	13 (5.5)	
Other physical activities	Weightloss *n* (%)	Increase muscle mass *n* (%)	Health and wellness *n* (%)	Rehabilitation *n* (%)	*p*
Yes	54 (31.2)	54 (31.2)	56 (32.4)	9 (5.2)	0.613
No	38 (39.2)	26 (26.8)	28 (28.9)	5 (5.2)	

*n* (participants); % (percentage); BMI (body mass index, kg/m^2^); Underweight (BMI < 18.5); Normal (BMI ≥ 18.5 and <25); Overweight (BMI ≥ 25 and <30); Obese (BMI ≥ 30); *p* (*p*-value from Chi-square test); University (university studies); No university (no university studies).

**Table 3:** Relationships between socio-demographic variables and sessions per week.

Age Groups	1 Session/Week *n* (%)	2 Sessions/Week *n* (%)	3+ Sessions/Week *n* (%)	*p*
18–34 years	26 (32.9)	45 (57.0)	8 (10.1)	0.456
35–49 years	52 (40.9)	62 (48.8)	13 (10.2)	
50–64 years	23 (41.1)	31 (55.4)	2 (3.6)	
65+ years	2 (25.0)	6 (75.0)	0 (0.0)	
BMI groups	1 session/week *n* (%)	2 sessions/week *n* (%)	3+ sessions/week *n* (%)	*p*
Underweight	1 (25.0)	2 (50.0)	1 (25.0)	0.179
Normal	59 (39.9)	73 (49.3)	16 (10.8)	
Overweight	36 (40.0)	48 (53.3)	6 (6.7)	
Obese	7 (25.9)	20 (74.1)	0 (0.0)	
Civil status	1 session/week *n* (%)	2 sessions/week *n* (%)	3+ sessions/week *n* (%)	*p*
Singles	38 (35.2)	58 (53.7)	12 (11.1)	0.324
Married	50 (37.3)	75 (56.0)	9 (6.7)	
Divorced/Widowers	15 (53.6)	9 (6.7)	2 (7.1)	
Level of studies	1 session/week *n* (%)	2 sessions/week *n* (%)	3+ sessions/week *n* (%)	*p*
No university	10 (31.3)	19 (59.4)	3 (9.4)	0.693
University	93 (39.1)	125 (52.5)	20 (8.4)	
Other physical activities	1 session/week *n* (%)	2 sessions/week *n* (%)	3+ sessions/week *n* (%)	*p*
Yes	69 (39.9)	92 (53.2)	12 (6.9)	0.407
No	34 (35.1)	52 (53.6)	11 (11.3)	

*n* (participants); % (percentage); BMI (body mass index, kg/m^2^); Underweight (BMI < 18.5); Normal (BMI ≥ 18.5 and <25); Overweight (BMI ≥ 25 and <30); Obese (BMI ≥ 30); *p* (*p*-value. Chi-square test); University (university studies); No university (no university studies).

## Data Availability

Not applicable.
